# Intraspecific and sex-dependent variation of leaf traits along altitude gradient in the endangered dioecious tree *Taxus fuana* Nan Li & R.R. Mill

**DOI:** 10.3389/fpls.2022.996750

**Published:** 2022-10-17

**Authors:** Tian-Xiang Li, Xiao-Lu Shen-Tu, Li Xu, Wei-Jun Zhang, Jun-Peng Duan, Yao-Bin Song, Ming Dong

**Affiliations:** Key Laboratory of Hangzhou City for Ecosystem Protection and Restoration, College of Life and Environmental Sciences, Hangzhou Normal University, Hangzhou, China

**Keywords:** altitude, endangered plant, intraspecific trait variation, leaf traits, sex-dependent variation, *Taxus fuana*

## Abstract

Plant intraspecific trait variation (ITV) including sex-dependent differences are matters of many ecological consequences, from individual to ecosystem, especially in endangered and rare species. *Taxus fuana* is an endangered dioecious species with small and isolated populations endemic to the Himalayas region. Little is known about its trait variation between sexes, and among populations. In this study, 18 leaf traits from 179 reproductive trees (males and females) along the altitude (2600-3200m a.s.l.) of the *T. fuana* populations distributed in Gyirong County, Tibet, China, were measured. ITV and sources of variation in leaf traits were assessed. The relationship between leaf traits of males and females and altitude was analyzed separately. Variations in leaf traits of *T. fuana* ranged from 3.1% to 24.2%, with the smallest in leaf carbon content and the largest in leaf thickness to area ratio. On average 78.13% of the variation in leaf traits was from within populations and 21.87% among populations. The trends in leaf width, leaf nitrogen to phosphorus ratio, leaf carbon to nitrogen ratio, leaf carbon isotope ratio, and leaf nitrogen isotope ratio in relation to altitude were the same for males and females. Leaf length to width ratio varied significantly with altitude only in males, while leaf phosphorus content, leaf nitrogen content, and leaf carbon to phosphorus ratio varied significantly with altitude only in females. The correlation coefficients of most leaf traits of females with altitude were larger than that of males. In the relationship between leaf traits, there was a high similarity among males and females, but the altitude accounted for more explanation in females than in males. Our results suggested that the variation in leaf traits of *T. fuana* was small and did not dominate the interspecific competition in the local communities. Adaptation to the altitude gradient of *T. fuana* might be through altering nutrient storage processes and water use efficiency. Adaptation of male and female *T. fuana* to environmental changes showed differences, where the males were more tolerant and the females responded greatly to altitude. The differences in adaptation strategies between male and female *T. fuana* may be detrimental to the maintenance of their populations.

## Introduction

The earliest study of plant intraspecific trait variation (ITV) could be attributed to the notable novelist and naturalist Johann Wolfgang von Goethe (Stegmann, 2021). However, most early studies in trait-based ecology focused on interspecific variation and only used a mean trait value per species, which assumed smaller trait variation among conspecific individuals (Suding et al., 2008; Shipley et al., 2016). Recent studies found that ITV of some plants could range from 10% to 40% of total variation (Albert et al., 2010; Messier et al., 2010; Siefert et al., 2015; Burton et al., 2017; Gaudard et al., 2019), 25% on average based on a global meta-analysis (Siefert et al., 2015). Another recent meta-analysis showed that the ecological effects of ITV are comparable to species effects (Des Roches et al., 2018). The ecological consequences of ITV have received ever more renewed interest from ecologists (Bolnick et al., 2011), such as population dynamics, interspecific interactions, stability, coexistence, and diversity of ecological communities (Steinmetz et al., 2020). Those findings suggest ITV could not be simply ignored in trait-based ecology (Shipley et al., 2016; Westerband et al., 2021; Rixen et al., 2022).

ITV is thought to be correlated with population size (Steinmetz et al., 2020), which means that populations of endangered species usually have lower ITV compared to widely distributed or common species. Species’ trait variation and covariation were thought to contribute to explaining ecological strategies in response to environmental gradients or changes (Jung et al., 2014). For instance, the variability of plant traits with altitude gradient reflects the adaptation of plants to the environment (Mathiasen and Premoli, 2016; de Villemereuil et al., 2018; Rathee et al., 2021). Changes in altitude lead to variations in some other environmental factors (e.g., temperature, light, etc.) that ultimately affect plant trait variability (Körner, 2007; Graae et al., 2012; Hartl-Meier et al., 2014; Sidor et al., 2015). Such trait–environment relationships are important for predicting the responses of global environmental change on individuals and populations of plants (Niinemets et al., 2015; Meng et al., 2017). Meanwhile, higher ITV could stabilize populations from extreme temporal fluctuations in population density and decrease extinction risk (Bolnick et al., 2011). This implies that exploration of ITV of rare and endangered species could contribute to understanding population demography, dynamic, and adaptation to natural and/or anthropogenic disturbances of rare and endangered species in changing environments (Meng et al., 2017; Song et al., 2020a). Therefore, incorporating ITV of rare and endangered species helps to better understand the mechanism for being endangered or threatened and benefits conservation practice (Chown, 2012; Cochrane et al., 2015; Turner et al., 2017; Álvarez-Yépiz et al., 2019). ITV generally includes morphological traits, functional traits, and stoichiometric traits by which to reveal the adaptation strategies of species to heterogeneous environments (Cordell et al., 2001; Elser et al., 2010; Albert et al., 2011; de Bello et al., 2011; Álvarez-Yépiz et al., 2019).

Sexual dimorphism is rather common not only in angiosperm but also in gymnosperm plants (64.6%) based on the latest estimation (Walas et al., 2018), such as Cycadidae, Gnetidae, *Ginkgo*, and some species in Pinidae (e.g. Taxaceae) (Ohri and Rastogi, 2020). The rate of dioecious plants is much higher than monoecious plants in the temperate climate zone (Walas et al., 2018). As for altitude, it has been found that the proportion of dioecious plants decreases with increasing altitude (Lin et al., 2020). Moreover, high altitude tends to exhibit male-biased sexes, while females are more abundant in lower altitude regions, because the relative abundance of environmental conditions is more favorable for the reproduction and survival of females (Ortiz et al., 2002; Juvany and Munné-Bosch, 2015; Retuerto et al., 2018). Due to different reproductive demands and selective pressures, male and female plants usually expressed different physiological, morphological, phenological, and reproductive traits (Obeso, 2002; Song et al., 2016). Studies found that female plants generally have higher photosynthetic rates than males owing to a compensation mechanism for reproductive costs (Obeso, 2002; Wu et al., 2021). In addition, females usually are smaller and grow more slowly than males (Cipollini and Whigham, 1994; Li et al., 2007; Seyedi et al., 2019; Zhang et al., 2019). Accumulating studies on functional sex-related trait differences of dioecious angiosperm plants (e.g. *Populus*) responding to stressful biotic and abiotic environmental factors have been conducted under controlled systems (Retuerto et al., 2018; Xia et al., 2020). However, few studies on trait-based ecology incorporated trait variation between genders (Galfrascoli and Calviño, 2020), especially for gymnosperm plants in natural ecosystems across environmental gradients (e.g. altitude).

*Taxus fuana* Nan Li & R.R. Mill (syn. *Taxus contorta*), is an endangered and dioecious gymnosperm endemic to the Western Himalayas region and grows as a tree or large shrub in the understory of mixed or *Pinus* forests along an altitude gradient ranging from 2600 to 3200 m in Southwest Tibet (China), Nepal, North India, and Pakistan (Shah et al., 2008; Song et al., 2020b). Due to its timber production, traditional medicinal uses, and commercial production of Taxol (i.e. the cancer-inhibitory alkaloid Paclitaxel) like other species of *Taxus*, this species also suffered from heavy anthropologically disturbances (Poudel et al., 2013; Song et al., 2020b). Previous studies found males have higher height, larger diameter, shorter needles, smaller leaf area, and stomata density than females but no differences between sexes in specific leaf area (SLA), leaf nitrogen, and carbon concentration in *T. baccata* (Iszkuło et al., 2009; Cedro and Iszkuło, 2011; Stefanović et al., 2017). However, to date, few studies focused on ITV and sex-dependent variation responding to environmental changes in natural ecosystems, and little is known if there are sexual dimorphisms in terms of leaf functional traits of *T. fuana*.

In this study, we sampled leaf materials and measured 18 leaf traits of *T. fuana* along an altitude gradient (from 2600 m to 3200 m a.s.l.) in Gyirong County, aiming to quantify the ITV and their environmental explanation. Specifically, we address the following scientific questions: 1) What are intraspecific variations (including sex-dependent variations) of leaf traits in *T. fuana*? 2) How do leaf traits of *T. fuana* vary along the altitude gradient? 3) Whether males trees respond differently from female ones along an altitude gradient?

## Materials and methods

### Study site and sampling

This study was conducted at Gyirong County (28°21′–28°29′ N, 85°13′–85°21′ E, 2600-3200 m a.s.l.), southwest of Tibet in China. The region is a subtropical mountain monsoon climate, with a mean average temperature of 8 - 11°C, and a mean annual precipitation of 800 mm. Based on our field survey, we observed 6 main (sub)populations with total individuals of more than 5000 which showed significantly male-biased populations in this area (Song et al., 2020b).

**Figure 1:**
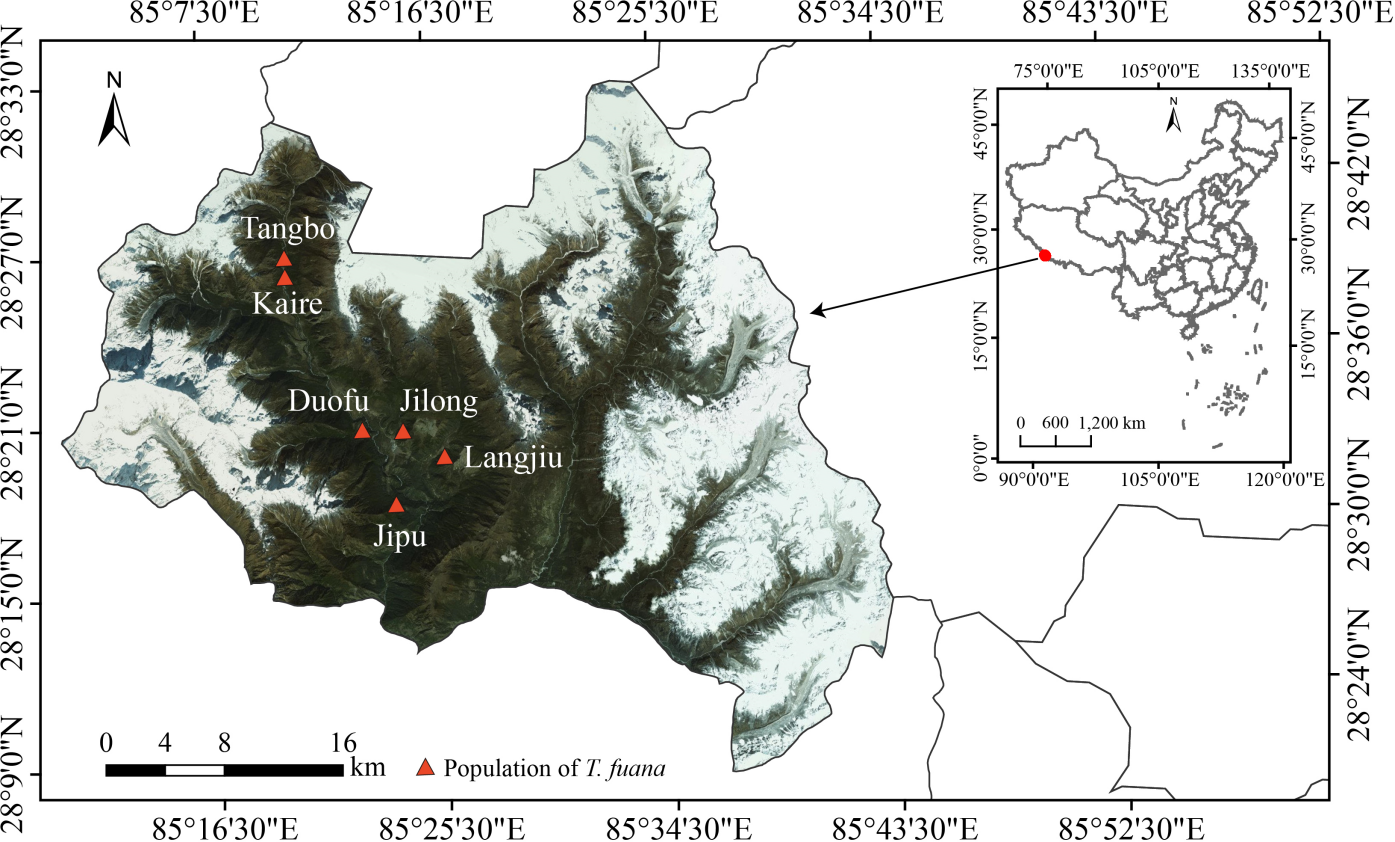
Geographical distribution of sampling sites (Li et al., 2022).

Among six known populations (Tangbo, Kaire, Guofu, Jilong, Langjiu, and Jipu) (Song et al., 2020b; Li et al., 2022) (**Figure 1**), individuals of *T. fuana* were randomly selected along an altitude gradient, with the distance between trees ensured to be above 50 m as far as possible. Fresh, mature (fully developed), and healthy leaves (needles) of *T. fuana* were sampled during the growing season (July–August) in 2018. In total, 179 samples were collected, of which 85 were males and 94 were females (The sex of the trees was identified according to whether they had obvious female cone or staminate strobilus). For each tree, we measured diameter at breast height (DBH), tree height, and altitude of the locality.

### Leaf traits measurement

A total of 18 leaf traits were measured and categorized into morphological traits, functional traits, and stoichiometric traits (Pérez-Harguindeguy et al., 2013). For morphological traits, 10 fresh leaves from each tree were randomly selected and the thickness of each leaf was measured with a vernier caliper (0.01 mm), which was used to calculate the mean leaf thickness (LT) (mm) of each tree. Then, those leaves of each tree were separately immersed in water overnight, blotted up water, and measured for water-saturated weight. After that, the same leaf samples were scanned with a photo scanner and weighed after oven-dried at 60°C for 72h. Leaf area (LA) (cm^2^), leaf length (LL) (cm), leaf width (LW) (cm), leaf perimeter (LP) (cm) of each sample were accessed from scanned photo with ImageJ (http://imagej.nih.gov/ij/), which also was used to determine leaf length to width ratio [leaf length (cm)/leaf width(cm)] (LLWR) (cm cm^-1^), leaf thickness to area ratio [leaf thickness (cm)/leaf area (cm^2^)] (LTAR) (cm cm^-2^) and leaf profile index [leaf perimeter (cm)/ $\sqrt{\text{LA}}$ (cm)] (LPI) (cm cm^-1^) (Pérez-Harguindeguy et al., 2013).

For functional traits, the specific leaf area (SLA) (cm^2^ g^-1^) of each sample was calculated as the ratio of sample leaf area to oven-dry weight. Leaf dry matter content (LDMC) (g g^-1^) was determined by the ratio of leaf oven-dry weight to water-saturated weight. Leaf carbon (C) and nitrogen (N) content (mg g^-1^) was determined using an elemental analyzer (vario MICRO cube; Elemental, Germany) (Hu et al., 2015). Leaf phosphorus content (P) (mg g^-1^) was determined by digestion with HClO_4_-HNO_3_ and then measured by Prodigy7 ICP-OES Spectrometer (Leeman, US) (Hu et al., 2019).

For stoichiometric traits, leaf carbon to nitrogen ratio (C:N), leaf carbon to phosphorus ratio (C:P), and leaf nitrogen to phosphorus ratio (N:P) were calculated by the ratio of leaf C to leaf N, leaf C to leaf P and leaf N to leaf P, respectively. Leaf carbon isotope ratio (δ^13^C) (‰) and leaf nitrogen isotope ratio (δ^15^N) (‰) were determined using the elemental analyzer coupled to an isotope ratio mass spectrometer (Isoprime100; Isoprime Ltd, Germany) (Hu et al., 2017; Hu et al., 2022; Tan et al., 2022).

### Statistical analyses

To describe the variation of *T. fuana* traits, the mean, minimum, maximum, and median values, as well as standard errors, and coefficients of variation (CV) were calculated for each leaf trait (Kumordzi et al., 2014). In order to analyze the intra-population variation of leaf traits, non-parametric multivariate analysis of variance with the “*adonis*” function in the “vegan” package was used to obtain among and within populations variance values for leaf traits, which were used as sources of variation among and within populations (Anderson, 2001). To investigate the overall response of *T. fuana* to environmental heterogeneity caused by altitude changes and the differential performance between males and females, a correlation analysis was conducted.

To explore the interrelationships among leaf traits of male and female *T. fuana*, and the differences between males and females, piecewise structural equation models (piecewiseSEM) were fitted to the leaf traits of male and female *T. fuana*, respectively (Lefcheck, 2016; Liu et al., 2020). Because the differences in light, water and heat conditions at different altitude (Körner, 2007; Moser et al., 2010) may have a greater impact on leaf traits of *T. fuana*, altitude was included as a random effect in the model, and the differences in marginal R^2^ (fixed factors only) and conditional R^2^ (all factors, including the random effect) were analyzed to assess the effect of altitude in *T. fuana* (Nakagawa and Schielzeth, 2013). Interrelationships among leaf traits were analyzed by standardized path coefficients. The fit of the model was confirmed using Fisher’s C test (when 0 ≤ Fisher’s C ≤ 2 and 0.05< *P* ≤ 1.00). The model was optimized by eliminating non-significant paths or factors (e.g., tree height, DBH, etc.), and the final model was obtained by comparison of models (Huang et al., 2020). The original model was constructed based on existing knowledge of the interrelationships of leaf traits (Navarro et al., 2010). A log_10_ transformation of the data was performed before model building. The main R packages involved in the model fitting process were “piecewiseSEM”, “nlme”, and “lme4” (Pinheiro et al., 2014; Lefcheck, 2016). All analyses were conducted using the statistical software R 4.2.1 (R Core Team, 2022).

## Results

### The variation of leaf traits in *T. fuana*

There was some variability among the 18 leaf traits of *T. fuana* (**Table 1**). The coefficients of variation were in the range of 3.1% to 24.2%. The larger variation occurred in leaf area, leaf thickness to area ratio, SLA, leaf P, and leaf C:P, respectively. The leaf thickness to area ratio was the most variable, ranging from 0.601-2.602, and the coefficient of variation was 24.2%. The smaller variation included leaf profile index, leaf C, and leaf δ^13^C, where the smallest variation was in leaf C, ranging from 460.674 to 527.157 and the coefficient of variation was 3.1%.

**Table 1:** Descriptive statistics of 18 leaf traits in *T. fuana* in six populations.

Leaf traits	Unit	N	Mean	SE	Min	Med	Max	CV (%)
LT	mm	179	0.481	0.004	0.372	0.477	0.642	10.6
LL	cm	179	2.730	0.031	1.658	2.720	4.255	15.3
LW	cm	179	0.197	0.002	0.121	0.196	0.255	11.0
LP	cm	179	6.228	0.068	3.886	6.204	9.520	14.6
LA	cm^2^	179	0.562	0.009	0.269	0.551	1.133	22.5
LLWR	cm cm^-1^	179	13.985	0.138	9.904	14.095	18.348	13.2
LTAR	cm cm^-2^	179	1.171	0.021	0.601	1.131	2.602	24.2
LPI	cm cm^-1^	179	8.337	0.033	7.301	8.303	9.330	5.3
SLA	cm^2^ g^-1^	179	79.680	1.332	40.738	77.826	141.937	22.4
LDMC	g g^-1^	179	0.358	0.003	0.279	0.356	0.474	10.1
Leaf C	mg g^-1^	179	499.408	1.147	460.674	503.608	527.157	3.1
Leaf N	mg g^-1^	179	14.880	0.195	8.294	15.011	21.852	17.5
Leaf P	mg g^-1^	179	1.595	0.028	0.844	1.524	3.493	23.7
Leaf C:N	–	179	34.640	0.486	23.126	33.529	58.770	18.8
Leaf C:P	–	179	328.896	5.437	141.577	327.732	609.256	22.1
Leaf N:P	–	179	9.564	0.123	5.844	9.560	14.572	17.3
Leaf δ^13^C	‰	179	−28.345	0.087	−31.613	−28.247	−25.282	−4.1
Leaf δ^15^N	‰	179	0.727	0.127	−2.913	0.632	5.498	232.9

Due to positive and negative values of leaf δ^15^N, there was a large degree of data dispersion, the CV of leaf δ^15^N was excluded from the analysis. LT, leaf thickness; LL, leaf length; LW, leaf width; LP, leaf perimeter; LA, leaf area; LLWR, leaf length to width ratio; LTAR, leaf thickness to area ratio; LPI, leaf profile index; SLA, specific leaf area; LDMC, leaf dry matter content; Leaf C, leaf carbon content; Leaf N, leaf nitrogen content; Leaf P, leaf phosphorus content; Leaf C:N, leaf carbon to nitrogen ratio; Leaf C:P, leaf carbon to phosphorus ratio; Leaf N:P, leaf nitrogen to phosphorus ratio; Leaf δ^13^C, leaf carbon isotope ratio; Leaf δ^15^N, leaf nitrogen isotope ratio.

The results obtained from non-parametric analysis of variance showed that 78.13% of the 18 leaf traits variation for *T. fuana* could be attributed to differences within populations, while the remaining 21.87% explained variation among populations (**Table 2**) (*F* = 9.684, *P* = 0.001). The results indicated that the variation in leaf traits of *T. fuana* mainly occurred within populations.

**Table 2:** Non-parametric analysis of variation for 18 leaf traits of *T. fuana* in six populations.

Source of variation	d.f.	Sum of squares	Mean of squares	Percentage of variation	*F*	*P*
Among populations	5	0.1221	0.0244	21.87	9.684	0.001
Within populations	173	0.4362	0.0025	78.13		
Total	178	0.5583				

### Patterns of variation in leaf traits with altitude in *T. fuana*

**Figure 2:**
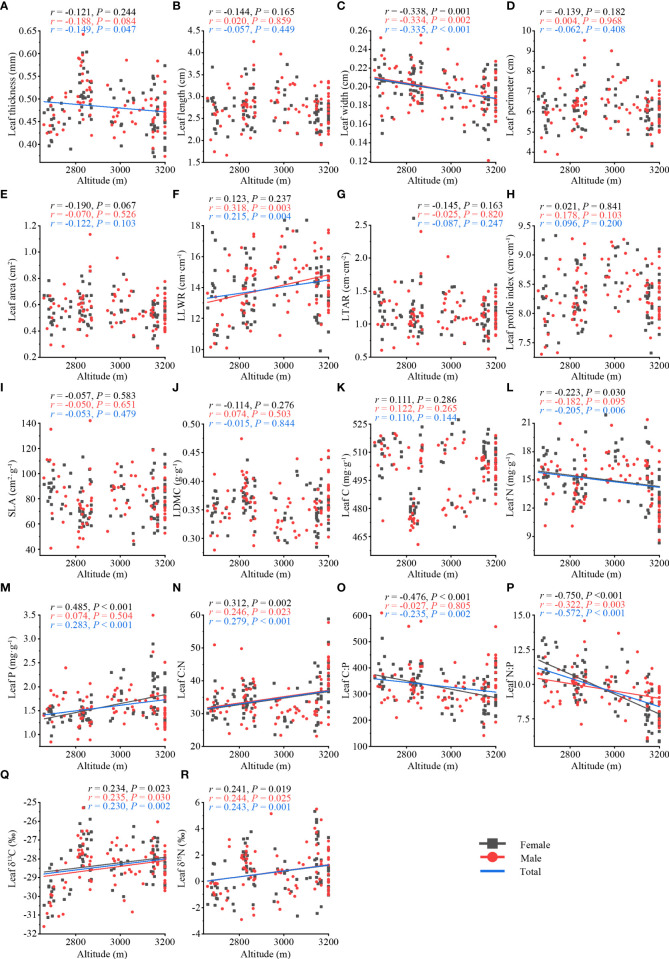
Relationship between leaf traits (total, female, male) along altitude in *T. fuana*. Morphological traits **(A–H)**, functional traits **(I–M)**, and stoichiometric traits **(N–R)**. Regression lines were plotted for significant relationships with *P* < 0.05. LLWR, leaf length to width ratio; LTAR, leaf thickness to area ratio; SLA, specific leaf area; LDMC, leaf dry matter content; Leaf C, leaf carbon content; Leaf N, leaf nitrogen content; Leaf P, leaf phosphorus content; Leaf C:N, leaf carbon to nitrogen ratio; Leaf C:P, leaf carbon to phosphorus ratio; Leaf N:P, leaf nitrogen to phosphorus ratio; Leaf δ^13^C, leaf carbon isotope ratio; Leaf δ^15^N, leaf nitrogen isotope ratio.

On the whole, the leaf thickness (*r* = −0.149, *P* = 0.047) (**Figure 2A**), leaf width (*r* = −0.335, *P*< 0.001) (**Figure 2C**), leaf N (*r* = −0.205, *P* = 0.006) (**Figure 2L**), leaf C:P (*r* = −0.235, *P* = 0.002) (**Figure 2O**), leaf N:P (*r* = −0.572, *P*< 0.001) (**Figure 2P**) of *T. fuana* were significantly negatively correlated with altitude, whereas the leaf length to width ratio (*r* = 0.215, *P* = 0.004) (**Figure 2F**), leaf P (*r* = 0.283, *P*< 0.001) (**Figure 2M**), leaf C:N (*r* = 0.279, *P*< 0.001) (**Figure 2N**), leaf δ^13^C (*r* = 0.230, *P* = 0.002) (**Figure 2Q**), leaf δ^15^N (*r* = 0.243, *P* = 0.001) (**Figure 2R**) were significantly positively associated with altitude. The remaining leaf traits were not significantly related to altitude (**Figures 2B, D, E, G–K**).

In terms of sex, the leaf width (female: *r* = −0.338, *P* = 0.001; male: *r* = −0.334, *P* = 0.002) (**Figure 2C**) and leaf N:P (female: *r* = −0.750, *P*< 0.001; male: *r* = −0.322, *P* = 0.003) (**Figure 2P**) of female and male *T. fuana* were significantly negatively correlated with altitude, whereas leaf C:N (female: *r* = 0.312, *P* = 0.002; male: *r* = 0.246; *P* = 0.023) (**Figure 2N**), leaf δ^13^C (female: *r* = 0.234, *P* =0.023; male: *r* = 0.235, *P* = 0.030) (**Figure 2Q**), and leaf δ^15^N (female: *r* = 0.241, *P* = 0.019; male: *r* = 0.244, *P* = 0.025) (**Figure 2R**) were significantly positively correlated with altitude in both female and male. However, the leaf length to width ratio (male: *r* = 0.318, *P* = 0.003) (**Figure 2F**) showed a significant increase with altitude only in males. In addition, leaf P (female: *r* = 0.485, *P*< 0.001) (**Figure 2M**) increased significantly with altitude only in females, and leaf N (female: *r* = −0.223, *P* = 0.030) (**Figure 2L**) and leaf C:P (female: *r* = −0.476, *P*< 0.001) (**Figure 2O**) decreased significantly with altitude only in females. It was worth noting that the number of leaf traits significantly correlated with altitude in females was higher than that in males, as well as the magnitude of correlation coefficients.

### Interrelations between male and female leaf traits in *T. fuana*

**Figure 3:**
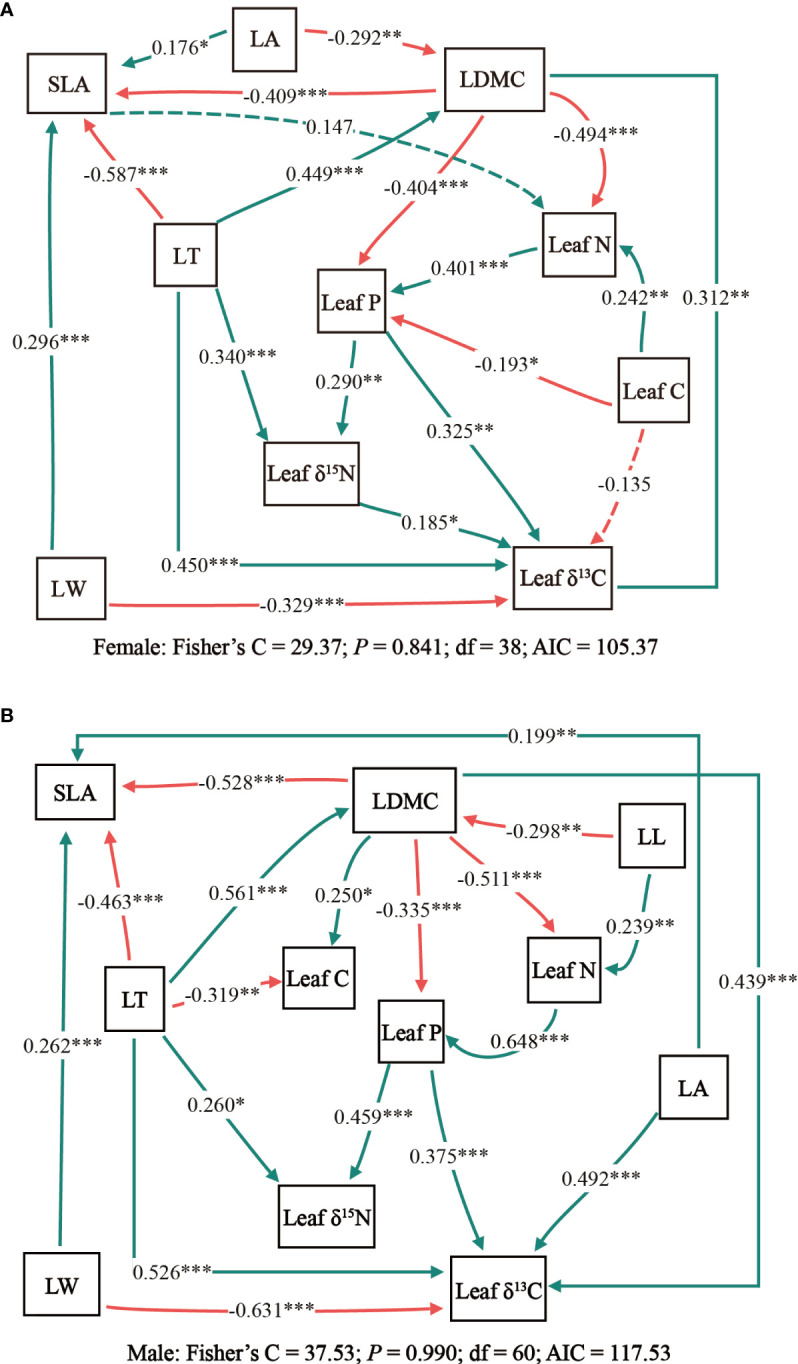
The piecewise structural equation models for testing the interrelations of leaf traits in female **(A)** and male **(B)** *T. fuana*. Solid arrows represent significant paths (*P*< 0.05) and dashed arrows represent non-significant paths (*P* > 0.05). The red arrows reflect negative relationships and the green arrows reflect positive relationships. Each path coefficient was standardized. *, **, *** indicated *P* < 0.05, *P* < 0.01, *P* < 0.001, respectively. LT, leaf thickness; LL, leaf length; LW, leaf width; LA, leaf area; SLA, specific leaf area; LDMC, leaf dry matter content; Leaf C, leaf carbon content; Leaf N, leaf nitrogen content; Leaf P, leaf phosphorus content; Leaf δ^13^C, leaf carbon isotope ratio; Leaf δ^15^N, leaf nitrogen isotope ratio.

Results of the structural equation model revealed that there was a greater similarity in the relationship between male and female leaf traits of *T. fuana* (**Figures 3A, B**). However, some differences were shown between the two models. As far as the direct effects of leaf traits are concerned, the principal variation in leaf traits between male and female *T. fuana* was manifested by the intervention of the additional leaf length and the relationship between leaf area and leaf C with other leaf traits in males. On the other hand, the path coefficients between leaf traits of male *T. fuana* were relatively larger than those of females.

The two structural equation models in this study differed significantly in the amount of explanation for leaf traits (**Table 3**). In female *T. fuana*, except for the LDMC, the conditional R^2^ (all factors) of the model for leaf traits was above 0.6. The variation of LDMC and leaf area explained by the model was minimum and maximum, respectively, and the conditional R^2^ (all factors) was 0.29 and 0.83. Correspondingly, the model of male *T. fuana* explained the variation of leaf δ^15^N was minimum, the conditional R^2^ (all factors) was 0.29. Among the other leaf traits, there were three leaf traits with conditional R^2^ (all factors) reaching 0.8, these were SLA, leaf P, and leaf δ^13^C. On the whole, the conditional R^2^ (all factors) of most leaf traits in male *T. fuana* was greater than that of females. Regarding marginal R^2^ (fixed effects), females had lower marginal R^2^ (fixed effects) than males except for leaf N. In contrast, the difference between marginal R^2^ (fixed effects) and conditional R^2^ (all factors) was higher in females than in males overall, which indicated that the random factor “altitude” accounted for more explanation in females than in males, namely, altitude had a higher effect on leaf traits in female *T. fuana* than in males.

**Table 3:** The marginal R^2^ and conditional R^2^ of the structural equation model in functional traits of female and male *T. fuana*.

Response variable	Female		Male	
	Marginal R^2^	Conditional R^2^	Marginal R^2^	Conditional R^2^
SLA	0.75	0.83	0.86	0.87
LDMC	0.22	0.29	0.34	0.47
Leaf P	0.46	0.69	0.69	0.80
Leaf C	–	–	0.08	0.72
Leaf N	0.47	0.67	0.41	0.57
Leaf δ^15^N	0.12	0.67	0.20	0.29
Leaf δ^13^C	0.46	0.64	0.49	0.80

Marginal R^2^ represents the variance explanation that includes only fixed effects, and conditional R^2^ represents the variance explanation that includes both fixed and random effects. “-” indicating the absence of marginal R^2^ and conditional R^2^ due to the elimination of paths to leaf C during the modification of the model. SLA, specific leaf area; LDMC, leaf dry matter content; Leaf C, leaf carbon content; Leaf N, leaf nitrogen content; Leaf P, leaf phosphorus content; Leaf δ^13^C, leaf carbon isotope ratio; Leaf δ^15^N, leaf nitrogen isotope ratio.

## Discussion

### Extent and sources of leaf trait variation in *T. fuana*

Generally, in most of the ecological studies on functional traits, the mean value of a trait was often taken to represent the whole species while ignoring ITV, which assumed there were few intraspecific variations compared to interspecific variations (Bolnick et al., 2011; de Bello et al., 2011; Shipley et al., 2016). However, numerous important functional traits showed considerable intraspecific variation with potential ecological effects similar to interspecific variation (Albert et al., 2011; Siefert et al., 2015; Moran et al., 2016; Laughlin et al., 2017; He et al., 2021; Rixen et al., 2022). The loss of ITV was even thought to be an important reason for its rareness in some wild species (Severns and Liston, 2008; González-Suárez and Revilla, 2013; Wang et al., 2022). Increasingly, studies found that ITV was probably an important ecological variable (Moran et al., 2016; Derroire et al., 2018; Mougi, 2020; Senthilnathan and Gavrilets, 2021), which played a non-negligible role in species coexistence (Ehlers et al., 2016; Hart et al., 2016; Zhang and Yu, 2018; Wang et al., 2022), interspecific competition (Kostikova et al., 2016; He et al., 2018; Xu et al., 2020), and population maintenance (Deepa, 2009; Bolnick et al., 2011; Raffard et al., 2019), etc. In this study, we found that leaf traits of six populations of *T. fuana* exhibited a certain degree of ITV, and the variation of leaf traits mostly originated from within populations and a small portion from among populations, which may be related to the distribution of *T. fuana* and local community features (Zhang et al., 2020; Li et al., 2022). It was shown that the importance of interspecific trait variation was likely to be greater than that of ITV at the large spatial scales of the study subjects. However, along with decreasing scales, for ITV, their relative importance would continually increase and possibly even emerge similar to that of interspecific trait variation (Messier et al., 2010; Albert et al., 2011; Kumordzi et al., 2014). Other studies had showed that the greater habitat heterogeneity, the more intraspecific and interspecific trait variation would be (Zhang and Yu, 2018; Homeier et al., 2021). The distribution area of *T. fuana* in the Gyirong region was relatively narrow (Song et al., 2020b), hence it showed a certain extent of ITV. Generally, the variation within populations reflects the plasticity and flexibility of the species to adapt to the environment, while the variation among populations reveals the influence of environmental selection (Siefert et al., 2015; Lajoie and Vellend, 2018; Moran et al., 2022). We found a small proportion of the ITV in *T. fuana* originated from among populations, perhaps because of the narrow distribution with little environmental heterogeneity among populations, and then failed to generate a large variation. In addition, the majority of trait variations originating from within populations may be associated with the position of *T. fuana* in the tree layer of the local community (Dan et al., 2020; Song et al., 2020b; Zhang et al., 2020). Plant communities in the Gyirong region were dominated by the tree layer, and strong interspecific competition led to a large plastic variation within the population. Therefore, the interactions between biotic factors in the community were perhaps the primary factors that determined the variation in *T. fuana* leaf traits.

### Variation patterns of leaf traits along altitude in *T. fuana*

Plant trait variation is often influenced by climate and topography (Brousseau et al., 2013; Heilmeier, 2019; Dobbert et al., 2021; Joswig et al., 2022), which in general is mainly influenced by climatic factors at the global scale and by topographic factors at the small scale (van Bodegom et al., 2014; Bruelheide et al., 2018; Myers-Smith et al., 2019; Shao et al., 2019; Zheng et al., 2022). Altitude is a key topographic factor (Graae et al., 2012; Waigwa et al., 2020; Tang et al., 2022), and changes in altitude can lead to changes in temperature (Sidor et al., 2015; Chen et al., 2018), precipitation (Luce et al., 2013; Herrmann et al., 2016), light (Körner, 2007; Thakur et al., 2019), etc., resulting in hydrothermal differences that affect plant traits (Hartl-Meier et al., 2014; Zhang et al., 2022). In this study, we found that the leaf traits of *T. fuana* showed different trends with altitude. Among them, leaf width became significantly smaller with altitude, while the leaf length did not change significantly with altitude, thus leading to a larger leaf length to width ratio with altitude. This indicated that the leaves of *T. fuana* were more needle-shaped with increasing altitude. It was also found that the leaf thickness decreased slightly with altitude, indicating that the leaves became softer while needle-shaped, which was probably related to the adaptation of *T. fuana* to snowfall and resistance to the tearing by strong winds. As the altitude increases, the likelihood and amount of snowfall become greater (Šípek and Tesař, 2014; Deng et al., 2017). The more needle-shaped and softer leaves would better avoid excessive snow accumulation and reduce damage, as well as resist extreme wind with some advantages (Read and Sanson, 2003; Onoda et al., 2011; Hua et al., 2020). In other aspects, the variation of leaf carbon content with altitude was not significant, thus the significant variation of leaf carbon to nitrogen ratio and leaf carbon to phosphorus ratio with altitude was mainly determined by the change in leaf nitrogen content and leaf phosphorus content, which indicated that the leaf carbon content of *T. fuana* was highly stable in accordance with universality (McGroddy et al., 2004; Elser et al., 2010; Yang and Luo, 2011). The leaf nitrogen content was reduced with increasing altitude, while the leaf phosphorus content showed the opposite trend, which in turn contributed to the reduction of leaf nitrogen to phosphorus ratio with increasing altitude. The leaf nitrogen content, leaf phosphorus content, and leaf nitrogen to phosphorus ratio reflect the nutrient utilization status of plants (Thompson et al., 1997; Reich and Oleksyn, 2004; Chen et al., 2013). The mean value of leaf nitrogen to phosphorus ratio in this study was 9.564, indicating that the growth and development of *T. fuana* were principally limited by nitrogen (Koerselman and Meuleman, 1996; Güsewell et al., 2003; LeBauer and Treseder, 2008). The decrease in nitrogen mineralization rate with increasing altitude probably inhibited the uptake of nitrogen by plants and thus caused a decrease in leaf nitrogen content (Reich and Oleksyn, 2004). In addition, the reduced plant growth and metabolic activities may also lead to lower leaf nitrogen content as the temperature drops with increasing altitude (Elser et al., 2003). In general, plants prefer to accumulate excess nutrients in extreme environments, especially excess leaf phosphorus content to enhance survival (Chapin, 1990; Cordell et al., 2001). Therefore, as the habitat was progressively harsher with increasing altitude, *T. fuana* might improve leaf phosphorus to adapt to the habitat change, especially under nitrogen limitations. This suggested that the environmental heterogeneity caused by altitude change affected the nutrient storage process of *T. fuana*. It was at the same time found that both leaf δ^13^C and leaf δ^15^N were greater with increasing altitude, which seemed to be related to the temperature and precipitation variability caused by altitude changes (Zhou et al., 2011; Yang et al., 2013; Li et al., 2017). In general, both temperature and precipitation were reduced with increasing altitude, and the reduction of temperature would diminish the diffusion capacity of CO_2_ in leaves, which in turn reduced the CO_2_ conductance of stomata and increased δ^13^C in leaves (Hultine and Marshall, 2000; Diefendorf et al., 2010). Meanwhile, leaf δ^13^C was an indicator of plant water use efficiency, and the higher the leaf δ^13^C, the higher the plant water use efficiency (Pascual et al., 2013; Barbour, 2017). This indicated that the water use efficiency of *T. fuana* gradually improved with increasing altitude and precipitation reduction, which was a manifestation that *T. fuana* adapted to environmental changes. The relationship between leaf δ^15^N and altitude was complicated, and the correlation differed with the species and environment in the studies (Liu and Wang, 2009; Gavazov et al., 2016; Wang et al., 2019). It has been shown that plant δ^15^N relates more to climate globally, such as negative relation with precipitation and positively correlated with temperature (Amundson et al., 2003). In this study, leaf δ^15^N was positively correlated with altitude, and from another perspective, it could be considered to be indirectly negatively correlated with temperature and precipitation, i.e., it is consistent with the relationship of precipitation in the global pattern and opposite to that of temperature. The possible reason was that the increase in altitude would lead to a decrease in precipitation and temperature, but the Gyirong region was more influenced by the warm and humid airflow from the Indian Ocean (Adhikari and Mejia, 2021; Li et al., 2022), so that precipitation might have a more prominent effect on *T. fuana*. Therefore, the leaf δ^15^N of *T. fuana* showed a positive trend with altitude. It implied that environmental factors are important drivers affecting the variation of *T. fuana* leaf traits, and their specific roles should be further considered in future studies.

### Interrelations and differences between male and female *T. fuana* leaf traits

Differences between dioecious plant traits and their response to environmental changes might detrimentally affect population maintenance (Melnikova et al., 2017; Hultine et al., 2018; LeRoy et al., 2020). In this study, leaf width, leaf nitrogen to phosphorus ratio, leaf carbon to nitrogen ratio, leaf δ^13^C, and leaf δ^15^N showed similar trends with altitude between male *T. fuana* and females, which reflected the similarity in adaptation to altitude changes between genders. Comparing the two structural equation models also exhibited such a situation, i.e., a greater similarity in the relationship between male and female leaf traits. This illustrated the coherence of adaptation strategies between male and female *T. fuana* (Ortiz et al., 2002; Munné-Bosch, 2015; Wang et al., 2018). However, in the correlation coefficients between leaf traits and altitude, females were mostly greater than males. Moreover, females exhibited remarkable performance in terms of stoichiometric characteristics. For instance, in leaf nitrogen content, leaf carbon to phosphorus ratio, and leaf phosphorus content, females reached significant levels with altitude only. Meanwhile, males showed relatively pronounced morphological characteristics, such as in leaf length to width ratio, where males reached significant levels with altitude only. These suggested that female *T. fuana* responded more strongly to altitude, and it can be inferred that the differences are mainly in nutrient utilization, while male *T. fuana* showed more stability to environmental heterogeneity caused by altitude changes (Lei et al., 2017). This might be related to the fact that males are more focused on vegetative growth, i.e. males may devote more resources to vegetative growth and thus have greater tolerance to corresponding environmental changes (Obeso, 2002; Meinzer et al., 2011). This was further illustrated in the performance of structural equation models, where the path coefficients among male leaf traits were mostly larger than the corresponding path coefficients for females (e.g., SLA, LDMC, leaf N, P, etc.). This may be related to the difference in resource absorption between the sexes. Studies showed that female trees allocated higher resources to reproduction than males, while males were likely to devote more resources to vegetative growth (Tognetti, 2012; Hultine et al., 2016; Lei et al., 2017). Therefore, this may result in the leaf traits of males being more closely linked and more tolerant to environmental changes, while females are more susceptible to multiple factors. A similar phenomenon was presented for the explained values (marginal R^2^ (fixed effect) and conditional R^2^ (all factors)). This suggested that the fixed factors could explain more variation in male *T. fuana* leaf traits than in females, while the random factor (altitude) accounted for a greater amount of explanation in females. Therefore, the inference seems to be that female *T. fuana* was more influenced by other factors or responded more strongly to environmental variability. Males, on the other hand, possess higher stability and could tolerate more environmental changes. This result supports previous studies that males were more adaptable than females and in a more favorable position in population development, which in turn possibly led to male-biased sex in *T. fuana* populations (Song et al., 2020b). Overall, it is inferred that male and female *T. fuana* exhibited greater similarity in their strategies for adapting to environmental changes, but there was also a considerable extent of variation between the male and female.

## Conclusions

The analysis revealed that the variance of *T. fuana* leaf traits was at a low level and did not dominate the interspecific competition in the local communities. Altitude was an important factor affecting the variation of *T. fuana* leaf traits. Adaptation of *T. fuana* to altitude gradient probably through altered nutrient storage processes and water use efficiency. The growth and development of *T. fuana* was restricted mainly by nitrogen, and probably adapted to the altitude change by modifying the nutrient storage process and water use efficiency. Male and female *T. fuana* showed differences in their strategies for adapting to environmental variability, where the male *T. fuana* was more tolerant and the females responded more strongly to altitude changes and were more influenced by the environment. The differences in adaptation strategies between male and female *T. fuana* may be detrimental to the maintenance of their populations, besides the environment was an important aspect affecting *T. fuana*, which deserved further consideration in future studies.

## Data availability statement

The original contributions presented in the study are included in the article/supplementary files. Further inquiries can be directed to Yao-Bin Song, ybsong@hznu.edu.cn or Ming Dong, dongming@hznu.edu.cn.

## Author contributions

Y-BS and MD contributed to the conception of the study. X-LS-T, T-XL, LX, W-JZ, J-PD performed the experiment and collected data. T-XL and X-LS-T analyzed the data. T-XL and Y-BS wrote the manuscript. All authors contributed to the article and approved the submitted version.

## Funding

This research was funded by the National Key Research and Development Program of China, grant numbers 2016YFC0503100; and the National Natural Science Foundation of China, grant number 31670429 and 31400346.

## Acknowledgment

We thank Yu-Lu Qin, Wang-Kai Shu, and Jun-Chao Ruan for their help in data collection.

## Conflict of interest

The authors declare that the research was conducted in the absence of any commercial or financial relationships that could be construed as a potential conflict of interest.

## Publisher’s note

All claims expressed in this article are solely those of the authors and do not necessarily represent those of their affiliated organizations, or those of the publisher, the editors and the reviewers. Any product that may be evaluated in this article, or claim that may be made by its manufacturer, is not guaranteed or endorsed by the publisher.
